# The Effects of Silica Nanoparticles on Apoptosis and Autophagy of Glioblastoma Cell Lines

**DOI:** 10.3390/nano7080230

**Published:** 2017-08-21

**Authors:** Rafał Krętowski, Magdalena Kusaczuk, Monika Naumowicz, Joanna Kotyńska, Beata Szynaka, Marzanna Cechowska-Pasko

**Affiliations:** 1 Department of Pharmaceutical Biochemistry, Medical University of Białystok, Mickiewicza 2A, 15-222 Białystok, Poland; mkusaczuk@wp.pl (M.K.); mapasko@gmail.com (M.C.-P.); 2 Institute of Chemistry, University of Bialystok, K. Ciołkowskiego 1K, 15-245 Białystok, Poland; monikan@uwb.edu.pl (M.N.); joannak@uwb.edu.pl (J.K.); 3 Department of Histology and Embryology, Medical University of Białystok, Waszyngtona 13, 15-269 Białystok, Poland; beataszynaka@gmail.com

**Keywords:** autophagy, apoptosis, glioblastoma multiforme, nanotoxicity, silica nanoparticles

## Abstract

Silica nanoparticles (SiNPs) are one of the most commonly used nanomaterials in various medical applications. However, possible mechanisms of the toxicity caused by SiNPs remain unclear. The study presented here provides novel information on molecular and cellular effects of SiNPs in glioblastoma LBC3 and LN-18 cells. It has been demonstrated that SiNPs of 7 nm, 5–15 nm and 10–20 nm induce time- and dose-dependent cytotoxicity in LBC3 and LN-18 cell lines. In contrast to glioblastoma cells, we observed only weak reduction in viability of normal skin fibroblasts treated with SiNPs. Furthermore, in LBC3 cells treated with 5–15 nm SiNPs we noticed induction of apoptosis and necrosis, while in LN-18 cells only necrosis. The 5–15 nm SiNPs were also found to cause oxidative stress, a loss in mitochondrial membrane potential, and changes in the ultrastructure of the mitochondria in LBC3 cells. Quantitative real-time PCR results showed that in LBC3 cells the mRNA levels of pro-apoptotic genes *Bim*, *Bax*, *Puma*, and *Noxa* were significantly upregulated. An increase in activity of caspase-9 in these cells was also observed. Moreover, the activation of SiNP-induced autophagy was demonstrated in LBC3 cells as shown by an increase in LC3-II/LC3-I ratio, the upregulation of *Atg5* gene and an increase in AVOs-positive cells. In conclusion, this research provides novel information concerning molecular mechanisms of apoptosis and autophagy in LBC3 cells.

## 1. Introduction

*Glioblastoma multiforme* (GBM) is the most frequently diagnosed and highly aggressive form of primary brain tumor [1]. The median survival time of GBM patients is less than 15 months [2]. Although multidisciplinary approaches of treatment, including maximal tumor resection and the combination of irradiation and conventional chemotherapy are applied, GBM is still associated with poor prognosis and remains incurable [3]. It is believed that two factors make GBM treatment extremely difficult. Firstly, the brain itself has limited capacity of regeneration, and secondly, GBM is extremely invasive and therapy-resistant [2,4]. Therefore, extensive efforts to develop new therapeutic strategies relying on selective destruction of cancer cells are currently being explored. One of the latest solutions in cancer treatment is the application of nanoparticle-based technologies.

Recent development of nanotechnology raised the need of intensive investigation of the cytotoxic effects of nanomaterials [5]. To date, the cytotoxicity of different nanoparticles (NPs) has been demonstrated in various in vivo and in vitro studies [6]. This cell-damaging property of nanoparticles has prompted a widespread quest of nanomaterials with possible application in cancer research. Given this, nanoparticles have already been used in controllable drug delivery [7,8], and theranostics [9].

Silica nanoparticles (SiNPs) are one of the most commonly used nanomaterials in biomedical research due to their certain benefits e.g.,: biocompatibility, large surface area for biomacromolecules loading, relative stability, and low production costs [10,11]. SiNPs have widely been explored as biosensors, biomarkers, cancer therapeutics, DNA or drug delivery systems, and additives for food and cosmetics [12]. However, their cytotoxic effects have also been reported [13].

To date, the mechanisms by which SiNPs induce cytotoxicity are not completely clear. Heterogeneity of physicochemical parameters of SiNPs, for example: size, shape, structure, and elemental constituents allow them to display multidirectional mechanisms of action in cancer cells [14]. The key mechanisms that seem to be connected with silica nanotoxicity include production of the reactive oxygen species (ROS), DNA destruction or aberrant aggregation of nucleoplasmic proteins [12,13,15,16]. These cellular disturbances caused by SiNPs lead primarily to the apoptotic death of damaged cells. Apoptosis plays a pivotal role in the control of tumor growth [17]. It has been demonstrated that SiNPs can trigger apoptosis through the activation of various apoptotic pathways [18,19]. The death receptor-mediated apoptosis of SiNPs-treated cells has been confirmed in vitro in A549 cell line [18]. Other reports emphasize the role of the mitochondrial pathway initiated after exposure to SiNPs [20,21]. It has been shown that treatment with SiNPs resulted in generation of oxidative stress and ROS production, which in turn led to apoptosis by intrinsic apoptotic pathway [21]. The dose-dependent upregulation of *caspase-9* and *-3* genes in A431 and A549 cell lines has been noticed [21]. Ahmad et al. have proven that proapoptotic *Bax* and *caspase-3* genes were upregulated, while the anti-apoptotic *Bcl-2* gene was downregulated in human liver HepG2 cell line [20]. In addition to apoptosis, in much research SiNPs-mediated necrotic cell death has also been reported [22,23,24]. Exposition of human umbilical vein endothelial cells (HUVECs) to SiNPs with diameters of 304 and 310 nm resulted in enhanced necrosis, while treatment of alveolar macrophages with SiNPs resulted in 80% of apoptosis and 20% of necrosis in these cells [22]. Additionally, Corbalan et al. showed that low [NO]/[ONOO–] ratio advisable increased nitroxidative or oxidative stress and is closely correlated with endothelial inflammation and necrosis [23].

Recently, autophagy has been identified as a novel mechanism induced in cells after exposition to nanoparticles. Autophagy can be triggered by a variety of microorganisms (bacteria, viruses) or parasites. Since NPs may present similar sizes to the microorganisms, they can possibly be perceived as a foreign bodies and cause autophagy activation [25,26,27]. Autophagy can be defined as a cytoprotective mechanism aiming at lysosomal degradation and recycling of proteins and damaged organelles, to maintain cellular homeostasis [27]. However, prolonged and uncontrolled autophagy may cause harmful cellular dysfunction and results in cell death [28]. A growing body of evidence suggests that deregulation of autophagy may also contribute to the toxicity evoked by SiNPs [26,27]. Duan et al. have demonstrated that SiNPs-induced autophagy and endotelial dysfunction in HUVECs cell line occur through the PI3K/Akt/mTOR signaling pathway [26]. The same research group has found that in HepG2 cells, the SiNPs-induced autophagy and autophagic cell death were triggered by ROS generation, suggesting that SiNPs can be a potential factor disrupting cellular homeostasis [27].

In light of the available data, cellular and molecular effects of SiNPs treatment seem to vary in size- and cell type-dependent manner. Although a considerable amount of data concerning SiNPs nanotoxicity exists, still little is known about the mechanism of SiNPs cytotoxicity in aggressive type of tumors such as GBM. In our study, we focused on highlighting the cellular and molecular effects responsible for the cytotoxicity of 5–15 nm SiNPs in human glioblastoma LBC3 and LN-18 cell lines. The mechanisms of apoptosis and autophagy, as two crucial processes of damaged cells elimination, were investigated.

## 2. Materials and Methods

### 2.1. Reagents

The Dulbecco’s modified Eagle’s medium (DMEM), containing glucose at 4.5 mg/mL with GlutaMax^TM^, trypsin-EDTA, penicillin, streptomycin and fetal bovine serum Gold (FBS Gold) were provided by Gibco (San Diego, CA, USA). Passive lysis buffer, ReliaPrep RNA Cell Miniprep System and luminescent Caspase-Glo 9 Assay were provided by Promega (Madison, WI, USA), and BCA Protein Assay Kit by Thermo Scientific (Rockford, IL, USA). Annexin V Apoptosis Detection Kit I, JC-1 MitoScreen Kit, APO-Direct Kit were product of BD Pharmingen^TM^ (San Diego, CA, USA). Sigma-Fast BCIP/NBT reagent, acridine orange, fumed silica dioxide amorphous powder 7 nm, silica dioxide spherical, porous nanopowder 5–15 nm and silica dioxide nanopowder 10–20 nm, 3-(4,5-dimethylthiazol-2-yl)-2,5-diphenyltetrazolium bromide and dichlorodihydrofluorescein diacetate were provided by Sigma (St. Louis, MO, USA). Polyclonal (rabbit) anti-human LC3 antibody and alkaline phosphatase-labeled anti-rabbit immunoglobulin G were provided by Cell Signaling Technology (Boston, MA, USA). High Capacity RNA-to-cDNA Kit was purchased from Applied Biosystems (Foster City, CA, USA).

### 2.2. Cell Cultures and Exposure to Silica Nanoparticles

The LBC3 cell line was developed from *glioblastoma multiforme* tissue taken from 56-year-old female patient subjected to surgical tumor resection, and was kindly given to us by Prof. Cezary Marcinkiewicz (Department of Neuroscience, Temple University, Philadelphia, PA, USA) [29]. The LN-18 and human skin fibroblasts (CRL1474) cell lines were provided by American Type Culture Collection (ATCC). The LBC3, LN-18 cells and fibroblasts were cultured in DMEM, supplemented with heat-inactivated, 10% (FBS Gold), streptomycin (100 μg/mL) and penicillin (100 U/mL). The cells were cultured in Falcon flasks (BD Pharmingen^TM^, San Diego, CA, USA) at 37 °C, 5% CO_2_ and 95% air in an incubator Galaxy S+ (RS Biotech, Irvine, UK). At approximately 70% confluence, cells were detached with 0.05% trypsin, 0.02% EDTA and counted in a Scepter cell counter (Millipore, Billerice, MA, USA). Next, 2.0 × 10^5^ cells were seeded in 2 mL of DMEM in six-well plates. In order to minimize the aggregation of SiNPs, prior to the experiments nanoparticles were dispersed in deionized water by a sonicator (Sonopuls, Bandelin, Berlin, Germany), on ice for 10 min (160 W, 20 kHz,). After 24 h incubation, DMEM was removed and replaced with DMEM containing SiNPs suspensions at three different sizes: 7 nm, 5–15 nm and 10–20 nm, at concentrations ranging from 12.5 to 1000 μg/mL. The LBC3 and LN-18 cells not treated with SiNPs served as the negative controls. Next, the cells were incubated for 24 and 48 h and retained for further analyses.

### 2.3. Cell Viability

Cell viability was measured according to the manner of Carmichael et al. using 3-(4,5-dimethylthiazol-2-yl)-2,5-diphenyltetrazolium bromide (MTT) [30]. The LBC3, LN-18 cells and fibroblasts, at a density of 2.0 × 10^5^ per well, were seeded in 6-well plates. After 24 h DMEM was removed and replaced with DMEM containing SiNPs suspensions at three different sizes: 7, 5–15 and 10–20 nm, at the concentrations ranging from 12.5 to 1000 μg/mL. The untreated LBC3 and LN-18 cells served as the negative controls. Then, the both cell lines were incubated with SiNPs for 24 and 48 h. The LBC3 and LN-18 cells were washed three times with phosphate buffer saline (PBS) and then incubated with 1 mL of MTT solution (0.25 mg/mL in PBS) in 5% CO_2_ incubator, at 37 °C, for 4 h. The DMEM was removed and 1 mL of 0.1 mol/L HCl in absolute isopropanol was added. The absorbance of converted dye in living cells was measured at the wavelength of 570 nm on an Infinite M200 microplate reader (Tecan, Salzburg, Austria). The viability of LBC3 and LN-18 cells as well as fibroblasts cultured with SiNPs was calculated as the percentage of the untreated cells. All the experiments were done in duplicates in at least three cultures.

### 2.4. Characterization of Silica Nanoparticles

The characterization of SiNPs in deionized water or DMEM at different time points was carried out using the electrophoretic light scattering technique on Zetasizer Nano ZS analyzer equipped with a 4 mW He-Ne laser (Malvern Instruments, Malvern, UK). The voltage selection and measurement duration were performed using default settings of the apparatus. The size of the SiNPs was measured by the Dynamic Light Scattering (DLS) technique. Zeta potentials were determined on the basis of electrophoresis experiment (Electrophoretic Light Scattering, ELS) of the sample and by measurements of the velocity of the nanoparticles using Laser Doppler Velocimetry (LDV) method. In order to minimize particles aggregation, before addition to deionized water or DMEM, the stock suspensions of SiNPs (0.1 mg/mL in deionized water) were homogenized through a sonicator (Sonopuls, Bandelin, Berlin, Germany), 160 W, 20 kHz, for 10 min. The final concentration of nanoparticles in individual media was 100 μg/mL.

### 2.5. Detection of Apoptosis and Necrosis

Apoptosis and necrosis of LBC3 and LN-18 cell lines were evaluated by flow cytometry on FACSCanto II cytometer (BD, San Diego, CA, USA). The cells (2.0 × 10^5^) were seeded in 2 mL of DMEM in six-well plates. After 24 h, the DMEM was removed, replaced with the 5–15 nm SiNPs suspension in DMEM, at 50 or 100 μg/mL concentrations. Both cell lines were incubated for 24 and 48 h. The cells were detached, resuspended in DMEM and then in binding buffer. Subsequently, the cells were stained with FITC Annexin V and PI (FITC Annnexin V apoptosis detection Kit I, (BD Pharmingen^TM^, San Diego, CA, USA) at room temperature, in the dark, for 15 min. Data were analyzed using FACSDiva software (BD Pharmingen^TM^, San Diego, CA, USA).

### 2.6. Intracellular ROS Detection

The level of intracellular ROS was determined using dichlorodihydrofluorescein diacetate (DCFH-DA) assay, (Sigma, St. Louis, MO, USA). After diffusion through the cell membrane, DCFH-DA is deacetylated by cellular esterases to a non-fluorescent compound, which is later oxidized by intracellular ROS into a fluorescent 2′,7′-dichlorofluorescein (DCF). The LBC3 cells (10 × 10^5^) were seeded in 200 μL of DMEM in 96-well black plates. After 24 h, DMEM was removed and the cells were stained with 10 μM of DCFH-DA in PBS at 37 °C, 5% CO_2_ incubator, for 45 min. Then, the dye was removed and replaced with the 5–15 nm SiNPs suspensions in DMEM, at 50 or 100 μg/mL concentrations and incubated for 24 and 48 h. The DCF fluorescence intensity was measured by Infinite M200 microplate reader (Tecan, Salzburg, Austria), at the excitation wavelength of 485 nm and the emission wavelength of 535 nm. The intracellular ROS generation in SiNPs-stimulated LBC3 cells was shown as the intensity of fluorescence of the DCF.

### 2.7. Mitochondrial Membrane Potential (ΔΨm) Analysis

The LBC3 cells (2.0 × 10^5^) were incubated in 2 mL of DMEM in six-well plates. After 24 h, DMEM was removed and replaced with the 5–15 nm SiNP suspensions in DMEM at the concentrations of 50 or 100 μg/mL and incubated for further 24 and 48 h. Then, the cells were detached and at 1 × 10^6^ cells per mL were suspended in PBS. Subsequently, disruption of the mitochondrial membrane potential in LBC3 cells was assessed using MitoScreen kit (BD, San Diego, CA, USA), following the manufacturer’s instructions. Briefly, supravital cells were washed and resuspended in PBS supplemented with 10 mg/mL the lipophilic cationic probe 5,5,6,6-tetrachloro-1,1,3,3-tetraethylbenzimidazolcarbocyanine iodide (JC-1). Then, the LBC3 cells were incubated at 37 °C for 15 min, washed and resuspended in PBS, and analyzed using flow cytometry (BD FACSCanto II, San Diego, CA, USA). The percentage of cells with disrupted mitochondrial membrane potential (MMP) was calculated using the FACSDiva software (BD, San Diego, CA, USA).

### 2.8. Caspase-9 Activity Analysis

The activity of caspase 9 was measured using luminescent Caspase-Glo 9 Assay (Promega, Madison, WI, USA) according to the manufacturer’s protocol. Briefly, the LBC3 cells were seeded in 200 μL of DMEM in 96-well white-walled culture plates (1 × 10^5^ cells per well) and incubated for 24 h. Next, the medium was removed and replaced with the 5–15 nm SiNP suspensions in DMEM, at the concentrations of 50 or 100 μg/mL and incubated for 24 and 48 h. Next, a 100 μL of the Caspase-Glo 9 reagent was added. The resultant luminescence was measured in a microplate reader (Tecan, Salzburg, Austria) and presented as relative light units (RLU).

### 2.9. RNA Isolation

Total RNA was purified using ReliaPrep RNA Cell Miniprep System (Promega, Madison, WI, USA) with DNase I treatment according to the manufacturer’s protocol. In order to assess the quantity and quality of the extracted RNA, spectrophotometric analysis was performed (NanoPhotometer, Implen, Munich, Germany). The concentration of RNA, as well as A260/280 and A260/230 ratios were measured.

### 2.10. Gene Expression Analysis

cDNA synthesis was performed using High Capacity RNA-to-cDNA Kit (Gibco, San Diego, CA, USA) following the producer’s indications. Briefly, 1 μg of purified total RNA was reversely transcribed in a reaction mixture containing oligo dT-16 primers, random octamers, dNTPs and MuLV reverse transcriptase (RT) in total volume of 20 μL. As a template for real-time qPCR reactions 2 μL of cDNA was used. Amplification of the product was done using 2xHS-PCR Master Mix SYBR A (A&A Biotechnology, Gdynia, Poland). Primer sequences for the following genes: *Bim*, *Puma*, *Noxa*, *Bax* and housekeeping *RPL13a* were as described in our previous papers [31,32]. Primer sequences for the *Atg5* gene were as described by Alirezaei et al. [33]. Additional evaluation of primer accuracy was performed using the Primer-BLAST software. The applied reaction parameters were as follows: initial denaturation at 95 °C for 3 min, followed by 40 cycles of 95 °C for 1 min, 58 to 62 °C for 30 s, and 72 °C for 45 s. To perform real-time qPCR assay the CFX Connect Real-Time PCR System (Bio-Rad, Hercules, CA, USA) was used. Reactions were run in triplicates and the quantification of gene expression was analyzed using the relative quantification method with modification of Pfaffl [34].

### 2.11. Transmission Electron Microscopy

The morphological changes in human LBC3 cells were evaluated by the transmission electron microscopy (TEM). The LBC3 cells (2.5 × 10^5^) were seeded in 2 mL of DMEM in six-well plates. After 24 h, the medium was removed and replaced by the 5–15 nm SiNPs suspensions in DMEM at the concentration of 50 μg/mL. Subsequently, the LBC3 cells were incubated for 24 and 48 h. After incubation, the cells were centrifuged (1000× *g*, 5 min), fixed in a mixture of 2.5% glutaraldehyde and 2% paraformaldehyde in 0.1 M cacodylate buffer (CB) at pH 7.0, at 4 °C, for 1 h, and taken up into the agar blocks. Then, samples were washed in CB at 4 °C, for 1 h, post-fixed in 1% osmium tetroxide in CB at 4 °C, for 1 h and next dehydrated through a graded series of ethanol and embedded in glycid ether 100 (Epon 812). Ultrathin sections were contrasted with uranyl acetate and lead citrate, mounted on nickel grids and evaluated in a transmission electron microscope OPTON 900 (Zeiss, Oberkochen, Germany).

### 2.12. Western Analysis

Cells were washed with cold PBS and solubilized in 100 μL per well of passive lysis buffer. The lysates from each well were centrifuged at 10,000× *g*, at 4 °C, for 10 min. Samples of lysates containing 20 μg of protein were subjected to SDS-PAGE, as described by Laemmli [35]. The 12% polyacrylamide gel and constant current (25 mA) were used. The proteins were transferred to nitrocellulose membranes and subsequently pre-treated with Tris-buffered saline (TBS) containing 0.05% Tween 20 (TBS-T) and 5% non-fat dry milk at room temperature, for 2 h. Membranes were probed with the primary polyclonal (rabbit) anti-human LC3 I/II antibody (1:1000) in 5% dry milk in TBS-T at 4 °C, for 16 h. Subsequently, the alkaline phosphatase-conjugated secondary antibody against rabbit IgG (whole molecule) at 1:2500 dilution was added in TBS-T with slow shaking for 1 h. The membranes were washed with TBS-T and exposed to Sigma-Fast BCIP/NBT reagent (Sigma, St. Louis, MO, USA).

### 2.13. Protein Assay

Protein concentration in cell lysates was measured by the method of Smith et al. using BCA Protein Assay Kit (Thermo Scientific, Rockford, IL, USA). Bovine serum albumin was used as a standard [36].

### 2.14. Fluorescent Microscopy Assay

The acidic vesicular organelles (AVOs) formation were visualized by the acridine orange (AO). An acidotropic dye is used as a marker and suggests the occurrence of autophagy in the analyzed cells. AO is a fluorescent dye that moves freely across biological membranes and stains the DNA and the cytoplasm bright green. In lysosomes and acidic organelles, acridine orange is protonated, forms aggregates and display bright red fluorescence [37].

The LBC3 cells (2.5 × 10^5^) were seeded in 2 mL of medium in six-well plates. After 24 h DMEM was removed and replaced the 5–15 nm SiNPs suspensions in DMEM, at concentrations of 50 or 100 µg/mL. Next, the cells were incubated for 24 and 48 h. After incubation, the cells were washed twice with PBS and stained with 1mL of the dye, 10 µM acridine orange, at 37 °C, for 10 min. Subsequently the cells were analyzed by fluorescence microscopy (Olympus CXK41, U-RLFT50, Tokyo, Japan) equipped with a tungsten 50 W lamp, a 490 nm band-pass blue excitation filter, a 500 nm diachronic mirror, and a 515 nm long pass barrier filter. Next, the medium with staining solution was removed, and the cell layer was washed with PBS and analyzed under a fluorescent microscope, at 200-fold magnification. The hundred cells per sample were examined by fluorescence microscopy, according to the following criteria: the AVOs-positive cells were determined by counting the number of cells with red signal from AVOs staining in cytoplasm in comparison to the number of cells without red signal from AVOs.

### 2.15. Statistical Analysis

Mean values from three independent experiments ± standard deviations (SD) were calculated. The data were statistically analyzed using one way-ANOVA followed by Tukey’s post hoc *t*-test analysis. The significant differences of means were determined at the level of * *p* < 0.05 or ** *p* < 0.001.

## 3. Results

### 3.1. The Effect of Silica Nanoparticles on Cell Viability

**Figure 1:**
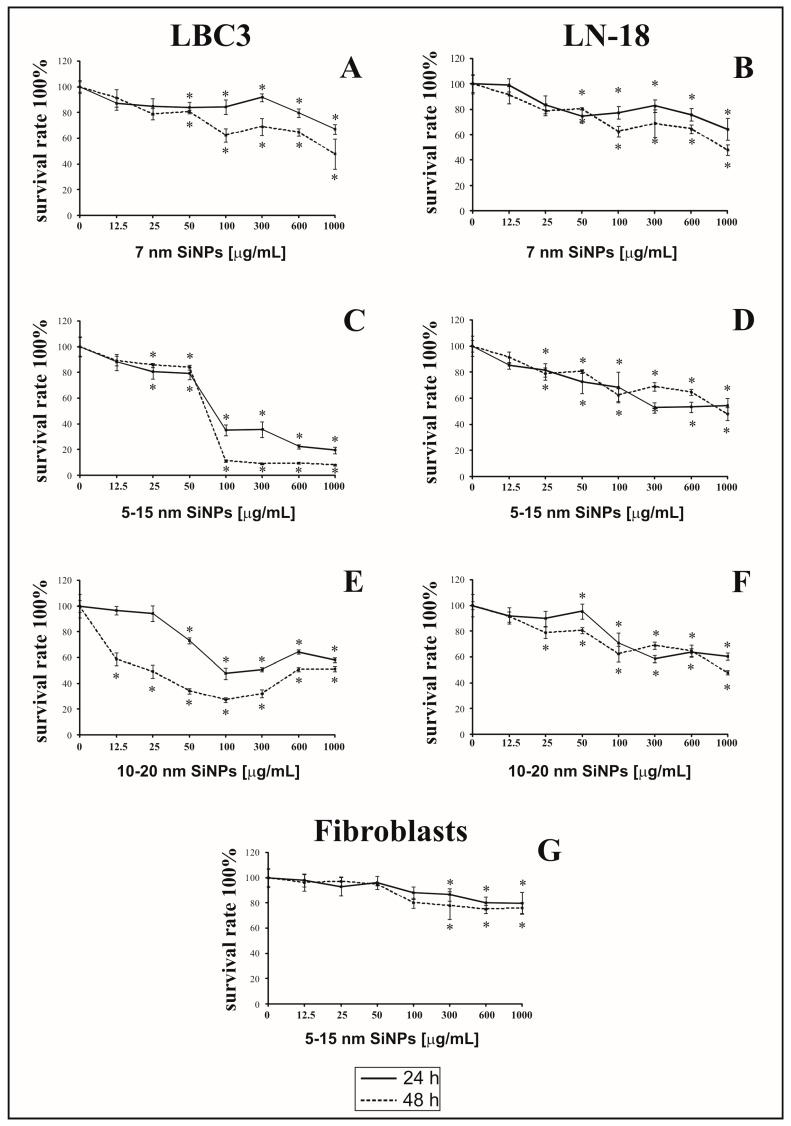
The viability of LBC3 (**A**,**C**,**E**) and LN-18 (**B**,**D**,**F**) cells treated with different concentrations (12.5 to 1000 μg/mL) of SiNPs in three different sizes: 7 nm (**A**,**B**), 5–15 nm (**C**,**D**) and 10–20 nm (**E**,**F**), for 24 and 48 h. Graph (**G**) presents the viability of human skin fibroblasts incubated with different concentrations, from 12.5 to 1000 µg/mL, of 5–15 nm SiNPs, for 24 and 48 h. Mean values from three independent experiments ± SD are presented. Significant alterations are expressed relative to controls and marked with asterisks. Statistical significance was considered if * *p* < 0.05.

The antiproliferative effect of 7 nm, 5–15 nm and 10–20 nm SiNPs on LBC3 (Figure 1A,C,E), LN-18 cells (Figure 1B,D,F) and human skin fibroblasts (Figure 1G) was determined using the MTT assay. The cells were incubated with increasing concentrations of SiNPs (ranging from 12.5 to 1000 μg/mL), for 24 and 48 h. It has been shown that SiNPs of all sizes caused time-dependent and dose-dependent reduction of cell viability in both, LBC3 and LN-18 cell lines. The reduction of cell viability was dependent on the sizes of SiNPs. The decrease in cell viability of LBC3 and LN-18 cells was observed after 24 h of incubation in all used SiNPs sizes. In cells treated with higher concentrations of SiNPs, the effect on cell viability was markedly more pronounced in case of the LBC3 cells; while in LN-18 cells the viability was much greater. Prolongation of incubation time up to 48 h, in cells incubated with SiNPs, resulted in strong decrease in number of viable LBC3 cells in comparison to viable LN-18 cells. The highest cytotoxicity was observed in case of cells treated with medium size SiNPs (5–15 nm) in both, LBC3 and LN-18, cell lines. The strong cytotoxic effect was observed after both time points of incubation with 5–15 nm SiNPs in LBC3 cells. In contrast to LN-18 and LBC3 cell lines we observed only weak, dose-dependent reduction in viability of normal skin fibroblasts using SiNPs 5–15 nm in the concentration from 12.5 to 1000 µg/mL (Figure 1G). Given this, based on the MTT results, 5–15 nm SiNPs in two concentrations: 50 and 100 µg/mL were selected for further studies.

### 3.2. The Effect of the Dispersion Media on Zeta Potentials

**Figure 2:**
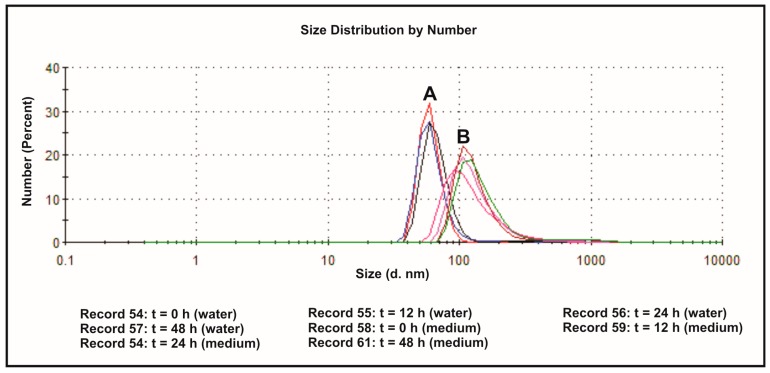
Size distribution of 5–15 nm SiNPs determined by DLS measurements: dispersed in deionized water (A), and dispersed in DMEM with 10% of FBS (B). Representative images generated by Zetasizer Nano ZS are presented.

The effect of the dispersion medium on zeta potentials and size distributions of SiNPs was measured in both deionized water and DMEM containing 10% FBS, at 37 °C and different time points. The values of the determined parameters have been collected in Table 1. Size distribution of SiNPs, dispersed in deionized water (A) and DMEM (10% of FBS) (B), has been presented on Figure 2. Zeta potential is the pivotal parameter that controls electrostatic interactions in nanoparticle dispersions [38]. The magnitude of the zeta potential is predictive of the colloidal stability. It is well known that higher absolute value of zeta potential reflects higher stable state of colloidal systems. The values of ζ-potentials higher than +30 mV or lower than −30 mV permits a basically stable suspensions, in which particles are not prone to aggregation or precipitation [39]. The ζ-potentials of SiNPs dispersed in deionized water show more negative values ranging from −32.6 to −35.1 mV compared to particles dispersed in culture medium (−8.11 to −8.96 mV). Silica nanoparticle sizes determined instantly after dispersion in water exhibited a bimodal size distribution profile, with one population (representing approximately 99.1% of all nanoparticles) with a size of 60.4 nm, and the other (representing about 0.9% of the nanoparticles) with a size of 329.5 nm (Table 1). These values changed slightly in time, showing higher percentage of ~400 nm aggregates after 12, 24 and 48 h (Table 1, Figure 2). The sizes of SiNPs measured instantly after dispersion in DMEM with 10% of FBS, exhibited a mono-disperse pattern with a peak at 133.1 nm. After 24 h, the particles showed a bimodal pattern with a peak at 129.9 nm (representing approximately 94% of all particles), and a peak at 673 nm (representing approximately 6% of all particles). Similar pattern of size distribution was observed after 48 h (Table 1, Figure 2). For all the particles, peaks in the large size area might represent agglomeration of particles. The sizes of SiNPs measured by DLS technique were larger than their original size, in both deionized water and DMEM with 10% FBS, which might be due to the van der Waals force and hydrophobic interaction with surrounding media.

### 3.3. The Effect of Silica Nanoparticles on Apoptosis and Necrosis

**Figure 3:**
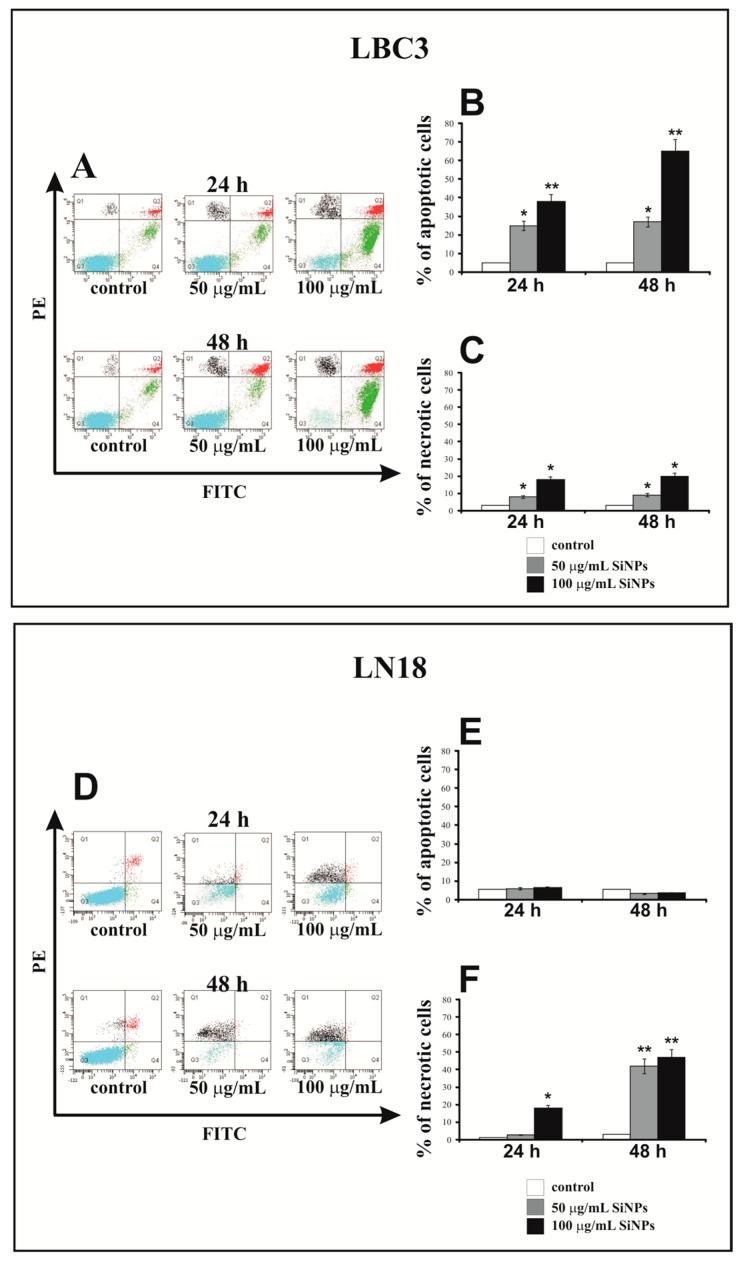
The effect of SiNPs on apoptosis (**A**,**B**,**D**,**E**) and necrosis (**A**,**C**,**D**,**F**) of LBC3 (**A**–**C**) and LN-18 (**D**–**F**) cell lines evaluated by annexin V assay. The cells were incubated for 24 and 48 h in DMEM with 50 and 100 μg/mL of 5–15 nm SiNPs. The cells were double-stained with FITC-Annexin V and PI. Representative dot plots for Annexin V-FITC/propidium iodide (PI) staining are shown (**A**,**D**). Following acquisition of sample, the cells were gated through the forward scatter FSC and side scatter SSC and analyzed for fluorescence intensity of FITC-Annexin V and PI. The cells were divided into four subpopulations: live cells—Q3 (annexin V-FITC−/PI−), early apoptotic cells—Q4 (annexin V FITC+/PI−), late apoptotic cells—Q2 (annexin V-FITC+/PI+), and necrotic cells—Q1 (annexin V FITC−/PI+). Percentage of apoptotic cells was the sum of percentage early apoptotic (Q4) and late apoptotic cells (Q2). Mean values of the percentage of apoptotic and necrotic cells, from three independent experiments ± SD are presented. Significant alterations are expressed relative to controls and marked with asterisks. Statistical significance was considered if * *p* < 0.05 or ** *p* < 0.001.

**Figure 4:**
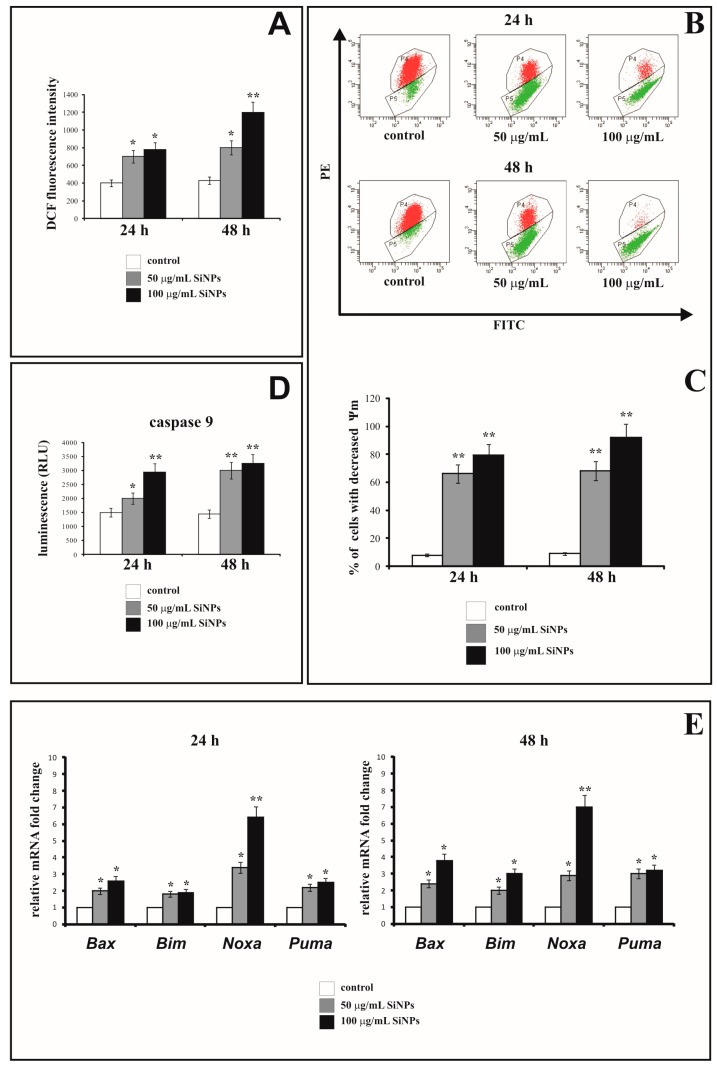
Oxidative stress and biochemical changes induced by 5–15nm SiNPs in LBC3 cells. The cells were treated with 50 and 100 μg/mL of 5–15 nm SiNPs for 24 and 48 h. Intracellular reactive oxygen species (ROS) production in LBC3 cells is presented on panel **A**. Panel **B** shows the flow cytometry analysis of ΔΨ_m_ in LBC3 cells. The top panel **B** shows representative dot plots of LBC3 cells analyzed by JC-1 staining. X-axis and Y-axis are green and red fluorescence, respectively. The gate P4—populations of cells with normal ΔΨm and gate P5—population of cells with decreased ΔΨm. The lower panel **C** shows the percentage of LBC3 cells with decreased ΔΨm. Panel **D** shows activity of caspase-9 in LBC3 cells. Relative quantification of proapoptotic genes expression in LBC3 cells is presented in panel **E**. Results are shown as a relative fold change in mRNA expression in comparison to untreated controls, where expression level was set as 1. Significant alterations are expressed relative to controls and marked with asterisks. Statistical significance was considered if * *p* < 0.05 or ** *p* < 0.001.

The apoptosis and necrosis of LBC3 and LN-18 cells were evaluated by flow cytometry on FACSCanto II (BD, San Diego, CA, USA). The percent of apoptotic and necrotic LBC3 cells incubated with 50 and 100 μg/mL of 5–15 nm SiNPs for 24 and 48 h is showed in Figure 3A–C. As depicted in Figure 3B, after 24 h of incubation in the cells treated with 50 and 100 µg/mL of 5–15 nm SiNPs, the percent of apoptotic cells was significantly higher in comparison to the control cells. We found a time- and dose-dependent increase in apoptosis of LBC3 cells. The percent of necrotic LBC3 cells in cultures incubated with 50 and 100 µg/mL of 5–15 nm SiNPs for 24 and 48 h, is reflected in Figure 3C. The dose-dependent but not time-dependent increase in necrosis of LBC3 cells was observed. The percent of apoptotic and necrotic LN-18 cells in cultures incubated with 50 and 100 µg/mL of 5–15 nm SiNPs for 24 and 48 h is showed in Figure 3D–F. In contrast to LBC3 cells, we did not observe any effect of SiNPs on apoptosis of LN-18 cells. The percent of apoptotic LN-18 cells did not change independently on the incubation time and doses of the SiNPs. In contrast to apoptosis, we observed the strong time- and dose-dependent effect of SiNPs on necrosis of the LN-18 cells (Figure 4D,F). In case of cultures incubated in DMEM with 50 µg/mL of 5–15 nm SiNPs for 24 h we did not observe changes in the percent of necrotic cells. In the cells incubated with 100 µg/mL SiNPs we observed a marked increase in necrosis in comparison to control cells. After 48 h of incubation of LN-18 cells with both concentrations of 5–15 nm SiNPs we noticed subsequent rise of necrosis in comparison to the control.

In LN-18 cells the primary mechanism initiated after treatment with SiNPs was necrosis, which is basically known as uncontrolled and passive process. Accordingly, to further focus on the mechanisms of SiNPs-mediated cell death, we have decided to continue our research on LBC3 cell line.

### 3.4. The Effect of Silica Nanoparticles on Intracellular ROS Generation

Figure 4A shows the fluorescence intensity of 2′,7′-dichlorofluorescein (DCF) in LBC3 cells incubated with 50 or 100 μg/mL of 5–15 nm SiNPs for 24 and 48 h. The fluorescence of DCF was intensified with an increase in the intracellular ROS production and was dependent on time of incubation and concentration of SiNPs. After 24 h-incubation of LBC3 cells with 50 or 100 μg/mL of 5–15 nm SiNPs, the intracellular ROS production was approximately 2-fold higher in comparison to the untreated cells. After 48 h, resulted in about 2-fold higher ROS production in LBC3 cells incubated with 50 μg/mL of 5–15 nm SiNPs, and 3-fold higher in case of cells treated with 100 μg/mL, in comparison to the untreated controls (Figure 4A).

### 3.5. The Effect of Silica Nanoparticles on the Change of Mitochondrial Membrane Potential

In order to evaluate the influence of SiNPs treatment on permeabilization of mitochondrial membrane, the assessment of the changes in mitochondrial membrane potential (ΔΨm) has been performed.

Figure 4B and C show the effect of 5–15 nm SiNPs on mitochondrial membrane potential (ΔΨ_m_) in LBC3 cells. Figure 4B shows representative dot plot of LBC3 cells stained with JC-1, and Figure 4C shows the percentage of LBC3 cells with decreased ΔΨ_m_. We observed the dose-dependent reduction in mitochondrial membrane potential in LBC3 cells. After 24 h as well as 48 h of incubation, a significant loss in ΔΨm was observed. The cells treated with 50 µg/mL of SiNPs showed approximately 10-fold decrease in ΔΨm, while cells incubated with 100 µg/mL of SiNPs showed nearly 11-fold reduction of ΔΨm in comparison to the control cells.

### 3.6. The Effect of Silica Nanoparticles on Caspase-9 Activity

Figure 4D shows the activity of caspase-9 in LBC3 cells exposed to 5–15 nm SiNPs at concentrations of 50 and 100 µg/mL, for 24 and 48 h. The activity of caspase-9 was increased in dose- and time-dependent manner. We observed 2-fold rising of caspase-9 activity after 48 h for both concentrations of SiNPs in comparison to the untreated cells.

### 3.7. The Effect of Silica Nanoparticles on Proapoptotic Genes Expression

The ROS generation can lead to simultaneous activation of apoptotic and autophagic cell death. The gene expression of *Bax*, *Bim*, *Noxa* and *Puma* in LBC3 cells exposed to 5–15 nm SiNPs at concentrations of 50 and 100 µg/mL for 24 and 48 h, was measured and results presented on Figure 4E. Our results showed that mRNA levels of *Bax*, *Bim*, *Noxa*, and *Puma* genes were upregulated in dose- and time-dependent manner in cells incubated with 5–15 nm SiNPs.

### 3.8. The Effect of Silica Nanoparticles on Morphological Changes of LBC3 Cells

**Figure 5:**
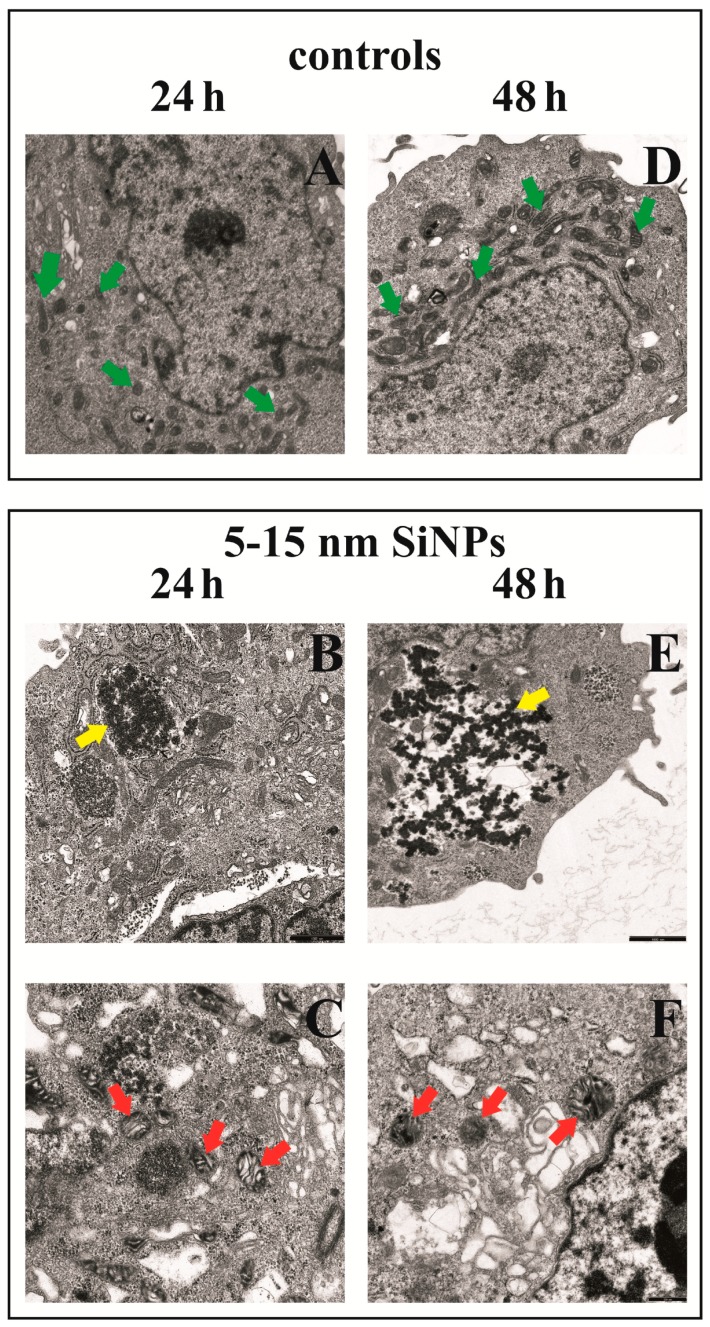
Morphological changes in LBC3 cells incubated with 50 µg/mL of 5–15 nm SiNPs for 24 (**A**–**C**) and 48 h (**D**–**F**) exposure. Control cells (**A**—left panel, **D**—right panel): numerous oval or rod-shaped mitochondria with clear intermembrane spaces were clearly visible (green arrows), (magnification 3000×). **B**—left panel and **E**—right panel: part of cell with electron dense regions located close to the membrane (yellow arrows), (magnification 7000×). **C**—left panel and **F**—right panel: a fragment of the cell with visible multiform mitochondria, mitochondrial swelling with firmly compacted mitochondrial matrix, bright spaces intermembrane (cristae), swelling and cristae rupturing (red arrows), are visible (magnification 12,000×).

Figure 5 shows the morphological changes of LBC3 cells exposed to 5–15 nm SiNPs (50 µg/mL). After 24 and 48 h of incubation, control cells had oval or rod-shaped mitochondria with medium or high electron density matrix (Figure 5A,D, green arrows). In LBC3 cells exposed to 5–15 nm SiNPs for 24 h we noticed destruction of mitochondrial structure such as focal brightening in the matrix, mitochondrial membranes deformation, mitochondrial swelling and cristae rupturing (Figure 5C, red arrows). Except that, as presented on Figure 5B, part of the cell is composed of a small electron dense material (SiNPs) either free or as membrane-bound aggregates in the cytoplasm (Figure 5B, yellow arrows). The changes in LBC3 cells exposed to 5–15 nm SiNPs for 48 h were similar to those observed in cells incubated with SiNPs for 24 h. Some alterations were more pronounced after 48 h. Changes in the mitochondria consist of altered size, shape and the focal edema with the damage to the mitochondrial membranes, mitochondrial swelling and cristae rupturing (Figure 5F, red arrows). Moreover, we observed a number of SiNPs dispersed in cytosol (Figure 5E, yellow arrows).

### 3.9. The Effect of Silica Nanoparticles on Expression of Autophagy Markers

**Figure 6:**
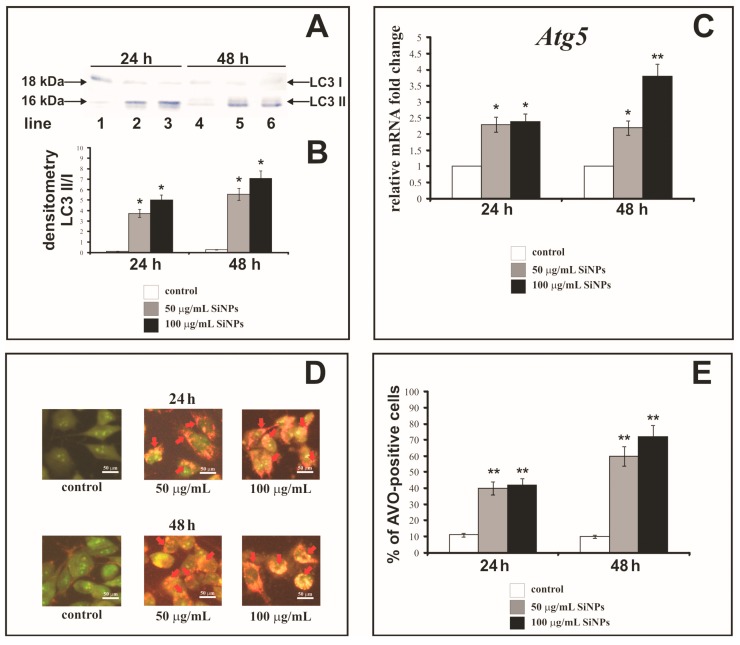
Western blot analysis (**A**) and densitometric analysis (**B**) of LC3-I and LC3-II ratio in LBC3 cells incubated in medium with 50 and 100 µg/mL of 5–15 nm SiNPs for 24 and 48 h. Samples containing 20 μg of protein were submitted to electrophoresis and immunoblotting. A representative Western blot is presented on panel A and densitometric analysis—on panel B. Relative quantification of *Atg5* gene expression in LBC3 cells (**C**). Results are shown as a relative fold change in mRNA expression in comparison to untreated controls, where expression level was set as 1. The effect of SiNPs on formation of AVOs in the LBC3 cell lines (**D**,**E**) evaluated by fluorescence microscope assay. The volume of the cellular acidic compartment was visualized by acridine orange staining. The cells were incubated in DMEM with 50 or 100 μg/mL of 5–15 nm SiNPs for 24 or 48 h and the cells were photographed under a fluorescence microscope at 200-fold magnification, (*scale bar* 50 μm) (**D**), or counted was the percentage of AVOs-positive cells (**E**). Representative images of AVOs positive cells (red arrows), from one of three independent experiments are shown. Significant alterations are expressed relative to controls and marked with asterisks. Mean values from three independent experiments ± SD are presented; * *p* < 0.05 or ** *p* < 0.001.

Figure 6A,B shows Western blot analysis of autophagy marker Light Chain 3 (LC3-I and LC3-II) expression in LBC3 cells incubated with 50 and 100 µg/mL SiNPs for 24 (lanes: 1, 2, 3) and 48 h (lanes: 4, 5, 6). The cells incubated for 24 and 48 h with 50 and 100 µg/mL of SiNPs demonstrate the expression of LC3-II form, and only week expression of LC3-I. Moreover, we observed that the expression of membrane-bound LC3-II, (the phosphatidylethanolamine-conjugated form) was increased in cells incubated with 50 µg/mL (lanes: 2, 5) and 100 µg/mL (lanes: 3, 6) of SiNPs after 24 and 48 h in comparison to the control cells (lanes: 1, 4). Next, we conducted densitometry analysis and calculated LC3-II/LC3-I ratio (Figure 6B). We noticed time- and dose-dependent increase of LC3-II/LC3-I ratio in cells incubated with both concentrations of SiNPs.

Figure 6C shows the level of *Atg5* gene expression in LBC3 cells exposed to 5–15 nm SiNPs at concentrations: 50 and 100 µg/mL, for 24 and 48 h. The transcript of *Atg5* was significantly upregulated in time- and dose-dependent manner in LBC3 cells (Figure 6C). Thus, we noticed the coexistence of increased LC3-II/LC3-I ratio with the upregulation of *Atg5* gene expression, confirming the occurrence of autophagy in cells subjected to SiNPs treatment.

### 3.10. The Effect of Silica Nanoparticles on Acidic Vesicular Organelles Formation

Autophagy can be characterized by the formation of AVOs (Acidic Vesicular Organelles), which can be detected by staining with acridine orange. This dye crosses the biological membranes and accumulates in acidic compartments, where it is seen as bright red fluorescence. Figure 6D shows AVOs formation in the cytoplasm, while Figure 6E shows the percent of AVOs-positive LBC3 cells after treatment with 50 or 100 µg/mL of 5–15 nm SiNPs, for 24 and 48 h. We observed a time- and dose-dependent (only after 48 h incubation) increase in AVOs formation in the cytoplasm of LBC3 cells. After 24 h of incubation, the percentages of AVOs-positive cells were about 4-fold higher in LBC3 cells treated with 50 µg/mL and 100 μg/mL of SiNPs, in comparison to control cells. The prolongation of the incubation time up to 48 h resulted in 6-fold higher percentage of AVOs-positive LBC3 cells when treated with 50 µg/mL of SiNPs, and 7-fold higher percentage of AVOs-positive cells, when treated with 100 µg/mL of SiNPs, in comparison to the control cells.

## 4. Discussion

A constant increase in number of currently diagnosed malignant diseases is calling for new treatment strategies involving the implementation of novel therapeutic approaches. Rapid progress in nanotechnology offers new solutions for development of new methods of cancer diagnosis and therapy. Biomedical research carried out in the last decade, showed that the chemical and physical properties of nanomaterials such as size or shape play an important role in determination for their cytotoxicity [40]. Furthermore, it is known that SiNPs are the most commonly used nanomaterials for medical applications because of the chemical stability, good biodistribution, cellular internalization and tumor penetration [21,41].

A great deal of research has demonstrated that SiNPs are cytotoxic and can cause ROS generation, DNA damage, aberrant nucleoplasmic protein aggregation, apoptosis and autophagy. These properties of SiNPs have prompted a widespread quest for the possible methods of their utilization as cancer therapeutics [42].

Moreover, preliminary reports suggest that SiNPs might be able to cross the blood-brain barrier, which makes them an interesting agent to use in glioblastoma research [43].

The charge, size and functionalization of NPs are important parameters determining the level of cellular damage of cancer cells. Our study has shown that SiNPs exerted cytotoxic effect on glioblastoma LBC3 and LN-18 cell lines but not in human skin fibroblasts. This cytotoxic effect in both glioblastoma cell lines was dependent on the size and dose of SiNPs as well as the time of incubation, which is in line with previous reports [44,45]. Kim et al. indicated, that the small-size SiNPs (20 nm) were more cytotoxic for U373MG human glioblastoma cells than the large-size ones (100 nm) [45]. Napierska et al. reported that in general, cells exposed to 5–15 nm SiNPs showed higher cytotoxic effect, than those exposed to 7 nm SiNPs, which is in agreement with our findings [46]. Similar results has been presented by Lin et al. who demonstrated that amorphous 5–15 nm SiNPs induced cytotoxic effect on human lung cancer cells [47]. Furthermore, Chang et al. demonstrated SiNPs-mediated cytotoxicity in lung, gastric and colon cancer cells as well as in normal fibroblasts [48]. Other authors concluded that the cytotoxicity of SiNPs was dependent on the cell type and population doubling time. Additionally, it has been proven that, intracellular distribution of SiNPs has a considerable influence on protein aggregation, gene expression and cell cytotoxicity [49]. Thus, SiNPs may penetrate cell membranes, lodge in the mitochondria, and lead to damage of cancer cells [49].

Physicochemical properties of SiNPs are important factors in nanotoxicity research [41]. We demonstrated that there were differences in *ζ*-potential values and nanoparticles sizes dependently on the dispersion medium. These differences are due to the adsorption of the proteins on the nanoparticle surface. It has been known, that the contact of NPs with biological fluids such as serum or ions, lipids, and other macromolecules is followed by instant adsorption of proteins on the nanoparticle surface in a form of a layer known as the protein corona [50]. Since DMEM contains 10% of FBS, this layer is likely to adsorb on the SiNPs surface. Protein coating may then alter the behavior of the nanoparticles, potentially modifying the aggregation state and cellular response [51]. Similar observations were also reported by other authors, indicating a constant need of empirical evaluation of physicochemical parameters of commercially available nanoparticles in particular experimental conditions [51,52].

The mechanisms of silica nanoparticles cytotoxicity are not fully understood and depend on their physicochemical properties such as: size [52]. Wittmaack [53] suggested that SiNPs can interfere with the membrane-mediated processes by gravitational settling of high concentrations of nanoparticles on top of the cells in the culture. On the other hand, it has been proven that SiNPs can induce oxidative stress and generate intracellular ROS formation. Mitochondria are involved in the generation of ROS through one-electron carriers in the respiratory chain. There are three major types of ROS, among others: superoxide anion (O_2_^−^), hydroxyl radical (HO·) and hydrogen peroxide (H_2_O_2_), which play pivotal role in cell metabolism, signaling, and homeostasis. Accumulation of ROS in the cells increases damage of the proteins, lipids, and nucleic acids [54]. Therefore, we decided to study the effect of SiNPs on ROS generation in LBC3 cell line. Our research showed that SiNPs induce intracellular ROS generation in LBC3 cells in dose- and time-dependent manner. Another in vitro studies have shown that nanoparticles caused oxidative stress, which impaired the balance between cellular ROS production and the mechanisms of ROS detoxification [41]. The increase in ROS has been suggested to be involved in apoptosis of cancer cells [20,21,55].

Apoptosis and necrosis are two characteristic types of cell death, which are discussed in many reports [17,31,32]. The two main mechanisms of the induction of apoptosis, the mitochondrial (intrinsic) pathway and receptor-mediated (extrinsic) pathway, are commonly known [56]. In our study, we evaluated the apoptosis and necrosis occurring in LN-18 and LBC3 cells after stimulation with SiNPs. Our research has shown that 5–15 nm SiNPs induces apoptosis and necrosis in LBC3 cells, whereas in LN-18 cells only necrosis occurs. The mechanism of necrosis is already well understood. Necrosis occurs as a result of a strong damage to the cell membrane that leads to rapid reduction of intracellular ATP levels and loss of osmotic balance of the cells [57]. Apoptosis however, can be induced by oxidative stress, and thus mitochondria play a pivotal role in this process [58]. We speculate that different survival rates between both cell lines exposed to the same nanoparticles sizes may be caused by different rate of endocytosis of SiNPs. We believe that, necrosis could be caused by a very fast up-take of SiNPs by LN-18 cells. There have been LN-18 cells overloaded with nanoparticles, which might results in the necrotic break down of these cells in comparison to LBC3, where the SiNPs overload was not that pronounced.

Mitochondria play pivotal role in activation of the intrinsic pathway of apoptosis in mammalian cells [59,60]. In the present study, we observed loss of the mitochondrial membrane potential and destruction of mitochondrial ultrastructure. In LBC3 cells in the presence of 5–15 nm SiNPs, we noticed mitochondrial swelling, cristae rupturing, deformation of mitochondrial membrane and over all mitochondrial damage. Due to the low MMP and changes in mitochondria ultrastructure, we suggest that 5–15 nm SiNPs-induced apoptosis in LBC3 cells occurs through the mitochondrial pathway. Ahamed showed that SiNPs generated oxidative stress and induced apoptosis by opening of the mitochondrial permeability transition pores (MPTP) and subsequent decrease of the MMP [58]. Moreover, during the opening of the MPTP, oxidative damage of mitochondrial membrane may occur due to the lower rate of hydroperoxide removal [58,59]. Sun et al. demonstrated that mitochondrial pathway of apoptosis mediated by oxidative stress was a potential mechanism of cytotoxicity induced by 43 nm SiNPs in HepG2 cell line [57]. Accordingly, we suggest that the loss of the MMP through the oxidative stress induced by 5–15 nm SiNPs, results in increased generation of ROS and further cytotoxic effect in LBC3 cells.

Cytochrome c is a protein, which upon extrusion from intermembrane space of mitochondria into the cytosol, forms a complex with apoptotic protease-activating factor 1 (APAF-1) and procaspase-9 and leads to the assembly of the apoptosome. These results in the activation of the caspase cascade, which evokes a series of biochemical and morphological alterations characteristic to apoptosis [59]. Therefore, we examined the activity of caspase-9 in LBC3 cells exposed to 5–15 nm SiNPs. Our results showed that capsase-9 activity increased in dose- and time-dependent manner. Nowak et al. demonstrated that SiNPs induced the expression of caspases-3 and -9 in cells [61], while Tokgun et al. found that SiNPs activated extrinsic pathway of apoptosis through the activation of caspase-8 in A549 cell line [18]. Apart from that, Ahmed showed that exposure of A431 and A549 cells to SiNPs, evoked upregulation of *caspase-3* and *caspase-9* genes in dose-dependent manner [21]. Moreover, the enzymatic activities of caspase-3 and caspase-9 were also enhanced in comparison to the control cells.

Next, we decided to investigate the main proapoptotic factors coupled with mitochondrial pathway of apoptosis at the molecular level. After 24 and 48 h treatment of LBC3 cells with 50 and 100 μg/mL of 5–15 nm SiNPs showed the upregulation of *Bim*, *Bax*, *Puma* and *Noxa* mRNA. Induction of apoptosis by SiNPs can be connected with transcriptional induction of BH3-only proteins such as PUMA, BIM, NOXA, BIK. The BH3-only proteins antagonize with anti-apoptotic BCL-2 family proteins e.g., BCL-2, BCL-xL, Mcl-1 and can activate the proapoptotic proteins BAX and BAK [23]. Activated BAX and BAK lead to the formation of pores in the outer mitochondrial membrane [17]. This suggests that the deregulation of transcription of genes involved in mitochondria-mediated apoptosis might play a pivotal role in the regulation of SiNPs-dependent death of LBC3 cells.

Most of the previous studies concerning SiNPs were focused on resolving the mechanisms of apoptotic and necrotic cell death. To date little is known about the toxicological consequences of SiNP-induced autophagy in cancer cells. Autophagy, also termed as type II cell death, plays an important role in cell survival during stress conditions. Paradoxically, this process can protect cancer cells, but may also contribute to cell death when the stress is prolonged and insuperable [27]. Autophagy involves the sequestration and degradation of cellular components, organelles or proteins by the lysosomal pathway [27]. It is controlled by a group of evolutionarily conserved genes (*Atg* genes) and it can lead to the induction, activation and nucleation of autophagic vesicles. During autophagy, double-membrane autophagosomes assemble in order to engulf intracellular components. The cytosolic form of microtubule-associated protein 1A/1B-light chain 3 (LC3-I) is conjugated with phosphatidylethanolamine during the process of autophagosomal membrane formation and subsequently generates LC3-II [62]. Conversion of LC3-I into LC3-II is commonly used to monitor autophagy and is known as the biomarker of this process. Furthermore, autophagy can be characterized by AVOs formation, ultrastructural analysis of autophagosomes [27]. Interestingly, SiNPs have been accepted as a new class of autophagy activators, since they could be recognized by cells as the endosomal pathogens or proteins, which are commonly degraded by autophagy pathway [27].

In our studies we observed an increase in LC3-II/LC3-I ratio, an upregulation of *Atg5* gene transcript, and an increase in AVOs-positive cells. Furthermore, we noticed the coexistence of autophagy and apoptosis in LBC3 cells exposed to 5–15 nm SiNPs. Recently, a few studies demonstrated that autophagy increased significantly after exposure to SiNPs [27]. Yu et al. showed that SiNPs could induce autophagy and autophagic cell death via the ROS generation in HepG2 cell line [27]. Apart from that, it has also been known that autophagy can induce apoptosis in ATG5-dependent pathway. ATG5 protein is one of the components of the basic autophagic machinery [63]. The key point in this mechanism is the cleavage of the ATG5 by calpain to create a cut form of the protein, that translocates into the mitochondria membrane, and cause the opening of the MPTP and activation of the intrinsic apoptosis pathway [63,64]. Additionally, another possible mechanism for autophagy-dependent cancer cell death is the selective recruitment of cell survival factors (including growth factors), for degradation in autophagosomes. Moreover, mammalian cells have been shown to selective by recruit cytoplasmic catalase to autophagosomes, which further leads to the accumulation of intracellular ROS and mitochondrial-dependent apoptosis [65].

## 5. Conclusions

**Figure 7:**
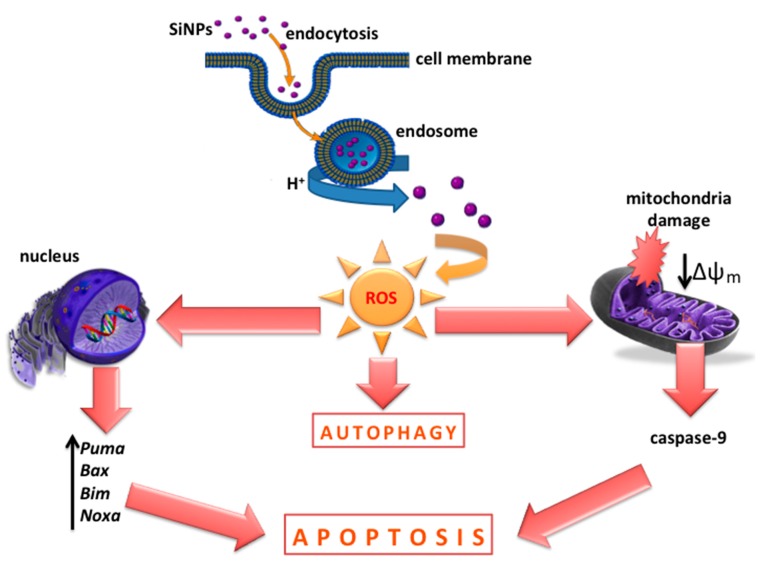
The effect of SiNPs on apoptosis, autophagy, and their suggested mechanism in glioblastoma LBC3 cells.

In conclusion, our results suggest that SiNPs can induce cytotoxicity in glioblastoma LBC3 and LN-18 cell lines, but not in human skin fibroblasts. Interestingly, we observed the coexistence of apoptosis and autophagy in LBC3 cells, while in LN-18 we noticed only necrosis. The SiNPs treatment resulted in oxidative stress and the loss of the MMP in LBC3 cells. Moreover, the upregulation of the proapoptotic genes: *Bim*, *Bax*, *Puma*, *Noxa* and increased activity of caspase-9 were observed in LBC3 glioma cells. These results may indicate that mitochondrial-dependent pathway of apoptosis is involved in SiNPs-mediated LBC3 cells death (Figure 7).

Although a great deal of information about SiNPs has already been available, it is still a subject of intensive investigations. Our findings demonstrate that SiNPs can act in a cell type-specific way and can initiate variable and complex mechanisms in response to their exposure. Therefore, it is worthwhile to comprehensively elucidate molecular mechanisms activated by SiNPs treatment to use it successfully as a potential therapeutic agent for glioblastoma multiforme therapy.

## Figures and Tables

**Table 1:** Zeta potential and size of SiNPs in deionized water (A) or DMEM with 10% of FBS (B) as dispersion medium at different time points (at 37 °C).

Times (h)	Deionized Water (A)			Medium (DMEM with 10% FBS) (B)		
	Zeta Potential (mV)	Diameter (nm)		Zeta Potential (mV)	Diameter (nm)	
		Fraction I	Fraction II		Fraction I	Fraction II
0	−32.6	60.14 (99.1%)	329.5 (0.9%)	−8.30	133.1 (100%)	-
12	−33.3	61.01 (97.5%)	269.2 (2.5%)	−8.24	140.0 (100%)	-
24	−35.1	62.05 (98.2%)	270.1 (1.8%)	−8.11	129.9 (94.0%)	673.0 (6.0%)
48	−35.0	65.93 (98.4%)	321.6 (1.6%)	−8.96	146.0 (95.9%)	893.0 (4.1%)

## Abbreviations

AVOs	acidic vacuolar organelles
DCFH-DA	dichlorodihydrofluorescein diacetate
GBM	glioblastoma multiforme
HUVECs	human umbilical vein endothelial cells
JC-1	5,5,6,6-tetrachloro-1,1,3,3-tetraethylbenzimidazolcarbocyanine iodide
LC3	microtubule-associated protein 1A/1B-light chain
MMP	mitochondrial membrane potential
MPTP	mitochondrial permeability transition pores
MTT	3-(4,5-dimethylthiazol-2-yl)-2,5-diphenyltetrazolium bromide
APAF-1	apoptotic protease activating factor 1
ROS	reactive oxygen species
SiNPs	silica nanoparticles
